# Editorial: Spotlight on allergy research in Asia

**DOI:** 10.3389/falgy.2024.1371795

**Published:** 2024-03-06

**Authors:** Kyoung Yong Jeong, Andreas Ludwig Lopata

**Affiliations:** ^1^ Institute of Allergy, Yonsei University College of Medicine, Seoul, Republic of Korea; ^2^ Australian Institute of Tropical Health & Medicine, James Cook University, Townsville, QLD, Australia; ^3^ Tropical Futures Institute, James Cook University, Singapore, Singapore

**Keywords:** allergen, allergy immunotherapy, Asia, localization, standardization

**Editorial on the Research Topic** Spotlight on allergy research in Asia

A lot of studies on allergic diseases are being conducted and great progress has been made in Asia. Notably, advanced technologies such as genomics, transcriptomics, proteomics, and bioinformatics have enabled researchers to investigate the immunological responses to the environments and biochemical characteristics of allergen sources. The goal of this Research Topic was to highlight the progress made in Asia and inspire researchers. However, we were not able to cover all aspects. More insights on the allergens of regional importance in Asia can be gained from recent collections with similar subjects (1–4).

In the current Research Topic, we summarized some aspects of microbiome study, allergen sensitization patterns, and immunotherapy in Asia. The house dust mite (HDM) microbiome study describes the origin of endotoxins in the mite extracts (Yi et al.). It provided potential insight into controlling endotoxins in the mite extracts. Characteristics of the allergenic molecule itself are important for the induction of allergic responses. However, the allergen matrix effect is also important for the allergenicity of allergenic proteins since allergens are not the only molecules introduced to the immune system. Therefore, molecular patterns derived from allergen sources need to be investigated in more detail.

HDMs and pollens are the most important causes of respiratory allergy in Asia. Oak, mugwort, ragweed, and Japanese hop are the most common causes of pollinosis in Korea. Mugwort in China and Japanese cedar in Japan are the most potent causes of pollinosis. In particular, three dust mite species (*D. farinae*, 59.8%; *D. pteronyssinus*, 50.4%; and *Blomia tropicalis*, 49.6%) seem to be prevalent sensitizers in Vietnam (Trinh et al.). Interestingly, *Blomia*-sensitized patients were older, and *Dermatophagoides*-sensitized patients were younger than non-sensitized groups. More in-depth studies are needed on the sensitization patterns to dust mite allergens. More studies are needed on the cockroach species and termites. Cockroach sensitization was investigated using German cockroaches but Oriental cockroaches are also found in Vietnam and other Asian countries. No information is available on termite species. Studies on the allergens from wheat, buckwheat, and soybean are being performed in Asia. Most food allergies (grain and fish) affect children and outgrow. However, wheat allergy and buckwheat allergy are common in adults. The allergenicity of traditional fruits in Asia is of interest. For example, Korean melon often causes pollen food allergy syndrome (PFAS) in Korea but no allergen has been identified yet. Many tropical fruits such as Jackfruit, avocado, lychee, papaya, passionfruit, guava, and mango, are commonly consumed in South Asia including Vietnam, Thailand, and the Philippines. However, the allergenicity of these fruits in Asia and their molecular characteristics are not well defined (5). PR-10 is thought to be mainly responsible for PFAS to tropical fruits. Studies on Asian vegetable allergy are also limited. For example, allergy to cilantro (coriander) is rare but commonly consumed in Asia.

Standardization of allergen extracts is inevitable for accurate allergy diagnosis and production of effective immunotherapeutics (6). Most of the reagents for the immunotherapy are imported from Western countries. The HDM is the first choice for local manufacturing in Asia. National reference materials were produced in Japan, Korea, China, and Thailand. Various reference materials supported by the government were produced in Korea, yet HDM extract is the only standardized extract that is commercially available (7). Japanese cedar in Japan and mugwort in China, which are allergen sources of regional importance, are the second choice for standardization.

We also can get some insight from a study from South Vietnam on the sensitization profile (Trinh et al.). The southern part of Vietnam is tropical and does not suffer from seasonal pollinosis. Sensitization patterns on pollen allergens are missing due to the lack of allergen extracts from indigenous plant species. In some cases, standardized pollen allergen extracts are not imported and the in-house preparations for skin tests are used. This implies the urgent necessity of collaboration with the manufacturers with GMP facilities and experienced researchers.

Yang et al. described some different situations of allergen immunotherapy in China partly due to the regulations of the Chinese government. It is necessary to localize more commercial products reflecting regional needs.

Not only differences in fauna and flora but also cultural differences between Western and oriental countries influence the allergen sensitization pattern. Food preferences and preparations are different among countries reflecting the cultural differences. The allergenicity of the foods depends on the cooking method. More studies are necessary on these aspects. Masumi et al. described the Japanese attitude toward reintroduction following an oral food challenge (OFC). This study provides insight into long-term outcomes after negative OFC. Eggs are not re-introduced into the children's diet largely due to the caregiver's anxiety, indicating the necessity of dietary guidance after OFC.

In-depth studies and progress on allergy research are being made in Asia. However, more interest should be given to differences from Western countries such as flora, fauna, culture, culinary preferences, and regulations. Especially, Asian fruit and vegetable allergens are of interest, and various allergens from foods indigenous to Asian countries are waiting for molecular characterization.
